# Unraveling SSc Pathophysiology; The Myofibroblast

**DOI:** 10.3389/fimmu.2018.02452

**Published:** 2018-11-13

**Authors:** Arjan van Caam, Madelon Vonk, Frank van den Hoogen, Peter van Lent, Peter van der Kraan

**Affiliations:** ^1^Experimental Rheumatology, Radboudumc, Nijmegen, Netherlands; ^2^Department of Rheumatology, Radboudumc, Nijmegen, Netherlands

**Keywords:** myofibroblast, systemic scleroderma, immune system, treatment, cytokine

## Abstract

Systemic sclerosis (SSc) is a severe auto-immune disease, characterized by vasculopathy and fibrosis of connective tissues. SSc has a high morbidity and mortality and unfortunately no disease modifying therapy is currently available. A key cell in the pathophysiology of SSc is the myofibroblast. Myofibroblasts are fibroblasts with contractile properties that produce a large amount of pro-fibrotic extracellular matrix molecules such as collagen type I. In this narrative review we will discuss the presence, formation, and role of myofibroblasts in SSc, and how these processes are stimulated and mediated by cells of the (innate) immune system such as mast cells and T helper 2 lymphocytes. Furthermore, current novel therapeutic approaches to target myofibroblasts will be highlighted for future perspective.

## Introduction

Systemic sclerosis (SSc) is a rare but severe auto-immune disease characterized by inflammation, vasculopathy and excessive fibrosis of connective tissues. Its incidence worldwide is on average an estimated 13 people per 1 million per year, with a prevalence of ~200 people per 1 million (1). Risk factors include genetic predisposition (2), female sex (3), and exposure to environmental cues such as chemicals like silica or solvents (4), but its etiology remains poorly understood. The excessive fibrosis characteristic for SSc typically starts distally in the skin of the extremities and moves upwards toward and trough the trunk until it greatly negatively affects the function of many organs like the gastro-intestinal tract and lungs. SSc is therefore accompanied by a high morbidity and patients often require extensive medical care with a (severely) reduced quality of life (5). Mortality is also increased in SSc patients. On average, the standard mortality rate of all causes is 2.7, with lung involvement being the major cause of death (6). Furthermore, the estimated loss of life-expectancy for patients is more than 15 years (7). Unfortunately, to date, no targeted disease-modifying therapy is available, resulting in a large unmet medical need. Because of this need, SSc has been designated an orphan disease to support research and development of a treatment.

The lack of targeted therapy for SSc is partly due to a lack of understanding of its pathophysiology. Its pathophysiology is a complex interplay between endothelium, the innate and acquired immune system, target organs and connective tissue which culminates in excessive fibrosis of e.g., skin and internal organs. A key cellular player in many fibrotic conditions such as keloid formation, Dupuytren's contracture and post-operative scarring is the myofibroblast, which is a special type of fibroblast. In this review we will discuss the role of myofibroblasts in SSc, their formation and how these cells are at the center of SSc pathophysiology, by regulating many of this disease's aspects.

## On the myofibroblast and its biological function

**Figure 1:**
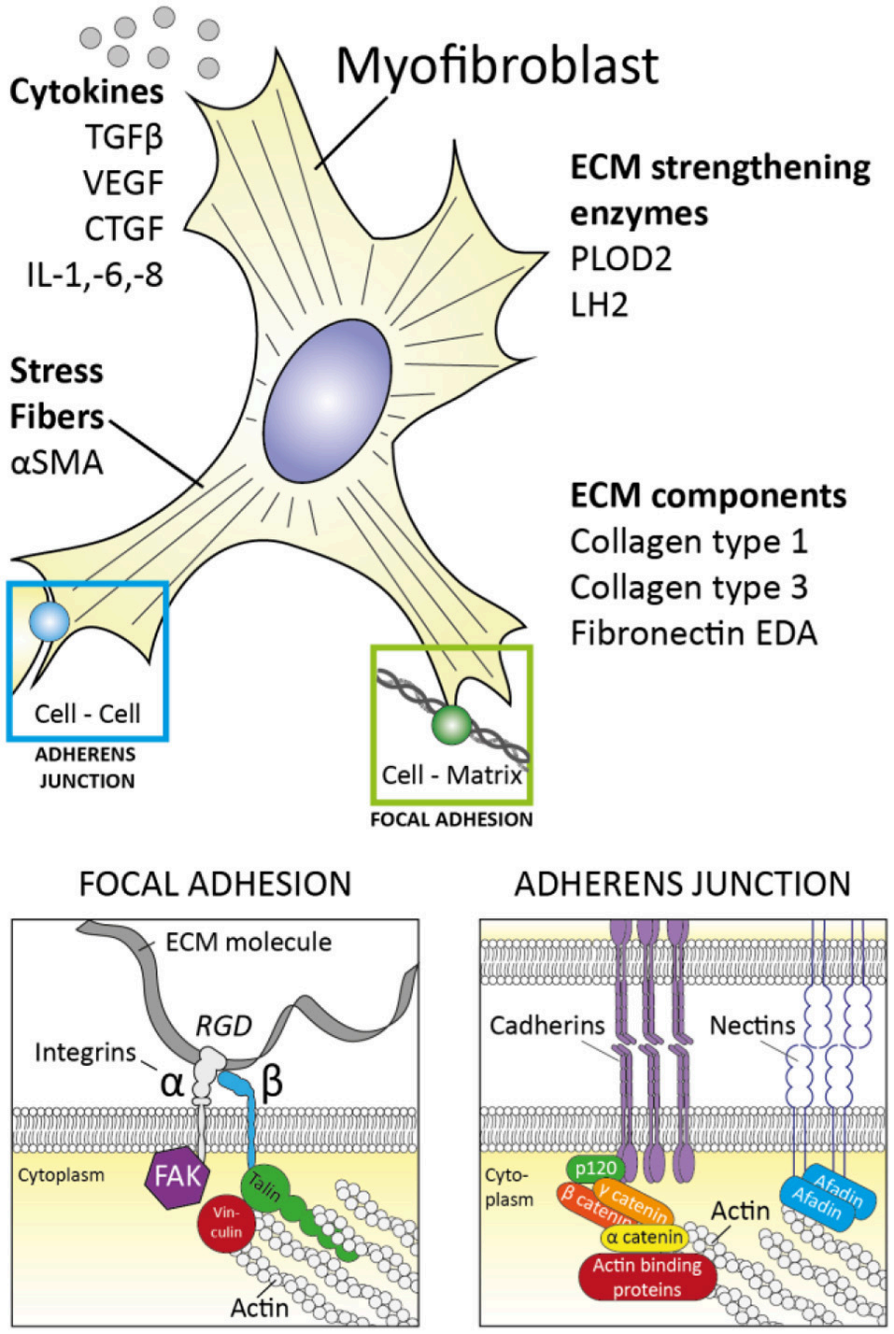
The myofibroblast and its properties. Myofibroblasts are characterized by stress fibers containing αSMA, production of extracellular matrix (ECM) components and ECM strengthening enzymes. Furthermore, myofibroblast are closely linked to their environment via focal adhesions and adherens junctions. Cytokines which are produced by myofibroblasts include TGFβ, VEGF, CTGF, IL-1, IL-6, and IL-8. These characteristics help myofibroblasts fulfill their role in wound healing.

Myofibroblasts were first identified in granulation tissue during open wound healing, as cells that resembled fibroblasts but contained microfilaments in their cytoplasm similar to those of smooth muscle cells (8, 9). Subsequently, it was demonstrated that these cells have contractile properties and are key in open wound closure (9). Myofibroblasts facilitate wound healing in several ways (Figure 1); First, they are capable of producing large amounts of extra cellular matrix (ECM) molecules such as collagen type I, collagen type III and fibronectin to replace lost ECM. Secondly, myofibroblasts are contractile. Their microfilaments (also known as stress fibers) consist of alpha smooth muscle actin (αSMA) and non-muscle myosin type II (10) and can contract in typical actin-myosin fashion, albeit rather slowly compared to muscle actin myosin filaments. Thirdly, myofibroblasts strongly connect physically to their environment; via integrin-mediated focal adhesions and cadherin-mediated adherens junctions their actin cytoskeleton is strongly anchored to their surrounding ECM and neighboring cells, respectively (11). The combination of this strong connection to the environment with their ability to contract allows myofibroblasts to exert tension on their surroundings and contract (damaged) tissue. This contraction decreases wound size and is crucial for open wound healing. Long term wound healing is further supported by myofibroblasts via their ability to strengthen the ECM; myofibroblasts express several protein and collagen crosslinking enzymes such as protein-glutamine gamma-glutamyltransferase 2 (=transglutaminase 2), protein-lysine 6-oxidase (LOX), and procollagen-lysine, 2-oxoglutarate 5-dioxygenase 2 (PLOD2) (12). These enzymes help strengthen e.g., fibrillar collagen bundles by post-translationally modifying collagen molecules, which results in increased crosslinking of these molecules in collagen networks during the maturation phase of wound healing. These crosslinks increase this networks' strength and prevents enzymatic degradation and thus strengthen the (scar) tissue.

Myofibroblasts also secrete and/or activate various autocrine and paracrine mediators to facilitate wound healing. For example, myofibroblasts produce vascular endothelial growth factor (VEGF) (13). This polypeptide growth factor is key in the formation of new blood vessels. Furthermore, myofibroblasts produce endothelin 1, a potent vasoconstrictor but also a factor which stimulates the formation of new myofibroblasts (14) and enhances their function in regard to collagen production and contractile properties (15). Myofibroblast function is also enhanced by their production of connective tissue growth factor (CTGF), a matricellular protein which stimulates e.g., their formation and collagen type I production. A key growth factor which is produced (13) and potently activated by myofibroblasts is transforming growth factor β (TGFβ) (16). This polypeptide growth factor is strongly pro-fibrotic and stimulates myofibroblast formation and activity. TGFβ is produced in latent form [bound by latency associated peptide (LAP) and latent TGFβ binding proteins (LTBP)] but can efficiently be activated by myofibroblasts via an integrin-mediated process (16, 17). Of note, TGFβ induces the expression of ET-1, CTGF, and VEGF in myofibroblasts, indicating that this growth factor lays at the heart of the expression of these factors. In addition, myofibroblasts can produce a range of various cytokines and chemokines to aid in the recruitment and facilitate the function of (innate) immune cells (13). Most notably, they produce interleukin 1 (IL-1), interleukin 6 (IL-6), interleukin 8 (IL-8), and monocyte chemoattractive protein 1 (MCP-1) (13).

Together these abilities make myofibroblasts well suited to facilitate wound healing.

## On the presence of myofibroblasts in SSc

Myofibroblasts have long been associated with SSc pathophysiology (18). Already in 1972 it was identified that fibroblasts obtained from SSc skin have a pro-fibrotic phenotype and produce more collagens than control fibroblasts (19). In 1990 it was confirmed using immunohistochemistry that fibroblasts of SSc patients near lesional areas in skin, esophagus, and lungs contain alpha smooth muscle actin (20) and are thus myofibroblasts.

In skin, the presence of myofibroblasts correlates with the amount of (hyalinized) collagen and skin parameters related to fibrosis such as tightness, hardness and stiffness, and does so more significantly than inflammation (21–23), supporting for a role of myofibroblasts in the pathogenesis of these clinical signs. This skin thickening and hardening can occur to such extent that it impairs movement of e.g., fingers. Furthermore, excessive matrix deposition leads to loss of tissue architecture such as sweat glands and hair follicles.

In lungs of SSc patients, the presence of myofibroblasts in the interstitial space can already be observed early during the fibrotic process (24), and with progression of interstitial lung disease they can ultimately also be observed in bronchoalveolar lavage liquid of SSc patients (25). The presence of pathological myofibroblasts greatly negatively affects lung function. Their matrix producing ability destroys alveolar architecture and increases interstitial space thickness, which both hamper respiration. Furthermore, the presence of myofibroblasts can induce stenosis; the abnormal narrowing of bloodvessels, and blood vessel narrowing is further enhanced by myofibroblasts' expression of ET-1, a potent vasoconstrictor. This hampers pulmonary blood flow, and as a consequence induces strain on the right heart ventricule.

Another location where myofibroblasts can be detected in SSc is in the esophagus and gastric wall of patients with severe fibrosis (26). Here, myofibroblast presence results in loss of muscle function, making these tissues unable to contract. As a consequence, gastric acid can flow into the esophagus, causing gastro-oesophageal reflux disease.

**Figure 2:**
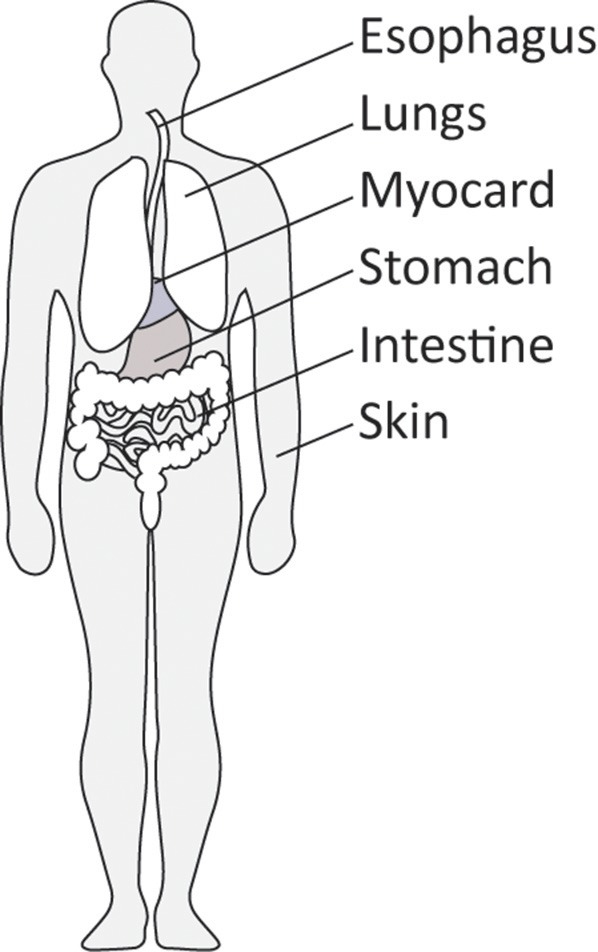
Organs commonly affected by diffuse cutaneous SSc.

Together, these observations place myofibroblasts in the various organs that can be affected by SSc. In addtion, organs such as kidney, intestine and myocard can also be affected by myofibroblast-driven fibrosis in SSc (18). However, of note, in late stage fibrotic atrophic SSc skin these cells can no longer be detected (27). Figure 2 gives an overview of the location of myofibroblasts in SSc.

In healthy tissues, the presence of myofibroblasts is (very) rare due to the tendency of myofibroblasts to undergo apoptosis when they are no longer needed for the healing process (28, 29). However, a putative resident type of myofibroblast can be found in lung alveolar ducts, where they help regulate alveolar function. In contrast, in SSc their presence is unwanted and attributed to a lowered susceptibility of myofibroblasts to undergo apoptosis and to increased formation.

## Decreased apoptosis of myofibroblasts in SSc

**Figure 3:**
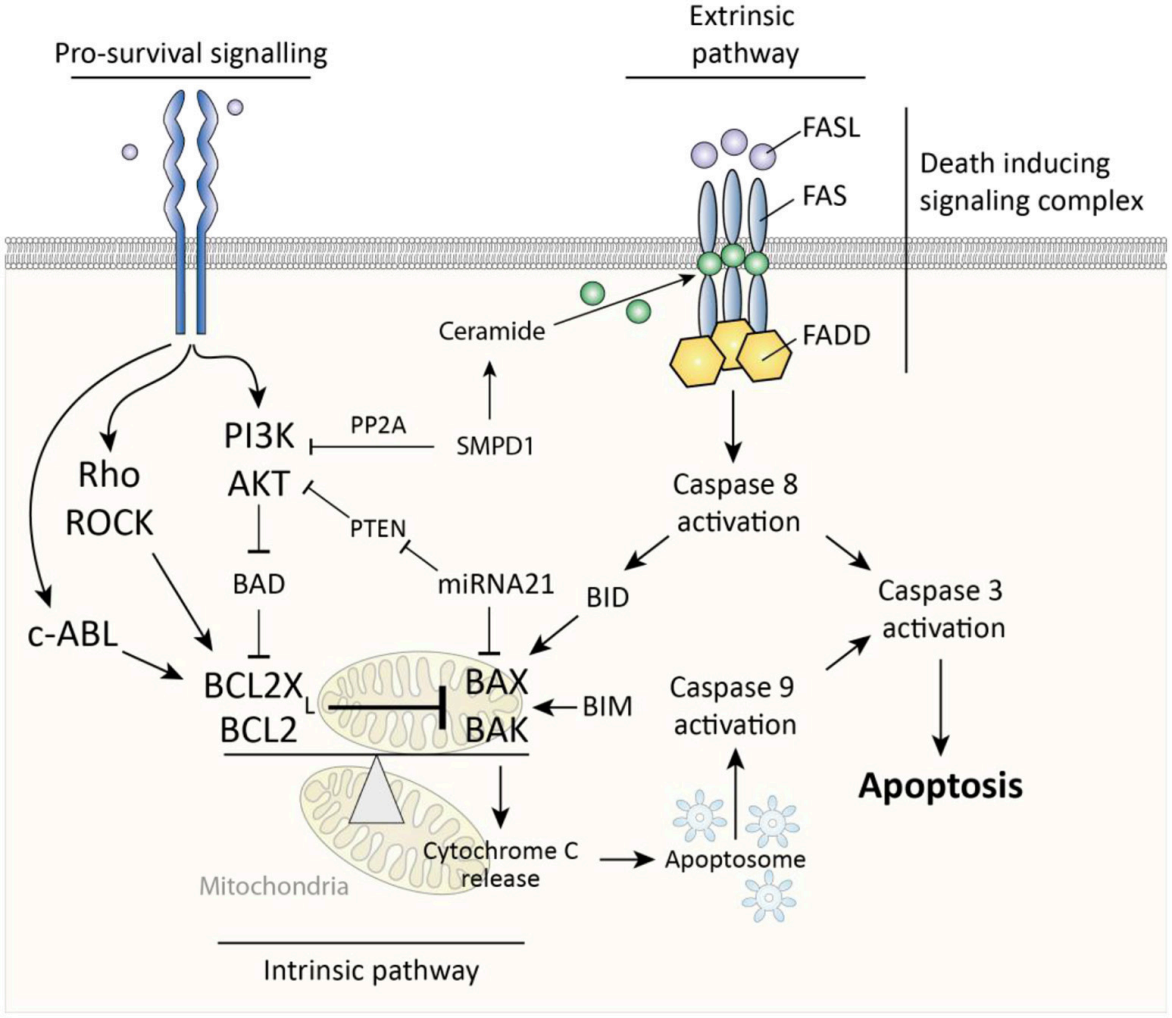
Caspase-dependent apoptosis pathways in myofibroblasts. The extrinsic pathway is activated via death inducing signaling complex and results in caspase 8-mediated caspase 3 activity which results in apoptosis. The intrinsic pathway is triggered by cytochrome c release from mitochondria which results in caspase 9-mediated caspase 3 activity. This cytochrome c release is governed by the ratio between pro-apoptotic BAX/BAK and BCL2(X_L_). Pro-survival signaling affects this ratio in favor of BCL2(X_L_).

Two major pathways govern cellular apoptosis; the intrinsic and extrinsic pathway. The extrinsic pathway is induced by activation of fas cell surface death receptor (Fas). Fas is a membrane spanning receptor of the TNF receptor superfamily and can, upon binding of Fas ligand, trigger the formation of a death-inducing signaling complex (DISC). This complex subsequently activates apoptosis-initiator caspase 8 to start a caspase pathway ultimately culminating in activation of caspase-3 and apoptosis (Figure 3). The intrinsic pathway is triggered by release of cytochrome c from mitochondria, which is subsequently incorporated into apoptosomes, cellular structures which activate the apoptosis-initiator caspase-9 to initiate apoptosis (30). A key protein in release of cytochrome c from mitochondria is BCL2-associated X protein (BAX), which, upon oligomerization, forms pores in the mitochondrial membrane through which cytochrome c can leak (31). Two important inhibitors of BAX are BCL2 and BCL2-X_L_ (also known as BCL2L1), which both prevent oligomerization of BAX and are thus anti-apoptotic. Of note, the extrinsic and intrinsic pathways are not fully discrete but linked, for example via BH3 interacting domain death agonist (BID), a protein which is activated by caspase 8 and subsequently forms mitochondrial membrane pores in cooperation with BAX (32). Ultimately, whether cells like myofibroblasts undergo apoptosis is determined by the ratio of activity between pro-apoptotic mitochondrial membrane pore forming proteins (e.g., BAX) and their anti-apoptotic inhibitors (e.g., BCL2). Pro-survival signaling can skew this balance in favor of anti-apoptotic proteins.

In systemic sclerosis, myofibroblasts are less prone to undergo apoptosis for several reasons. To begin, it has been observed that, in quiescent state, SSc myofibroblasts express less pro-apoptotic BAX compared to myofibroblasts of control subjects (33). A possible cause for this is increased activity of tyrosine-protein kinase ABL1 (c-Abl). Silencing of c-ABL enhances apoptosis in both healthy and SSc skin fibroblasts by increasing the BAX/BCL2 ratio toward pro-apoptotic BAX (34). An example of how c-ABL can be activated is via TGFβ signaling; in idiopathic pulmonary fibrosis, c-Abl is activated by TGFβ (35), and silencing of c-Abl inhibits the pro-survival effects of TGFβ on myofibroblast apoptosis (34).

Secondly, in fibrotic tissues, extracellular matrix stiffness is increased compared to healthy tissue. This increased stiffness is an important survival signal for myofibroblasts; via mechanosensing such stiffness results in intracellular activation of Rho and Rho-associated kinase (ROCK) whose activity increases BCL2-X_L_ expression (36). Importantly, this increased, stiffness-induced, BCL2-X_L_ expression is needed to counteract the function of the pro-apoptotic protein BIM (36). BIM is an activator of BAX and accumulates in myofibroblasts exposed to a stiff matrix. This accumulation primes the cells to undergo apoptosis (36), and only the continued presence of BCL2-X_L_ prevents this. This balance between BCL-2 and BIM serves a role during normal wound healing; once the matrix softens during the final wound remodeling stage, pro-surivival ROCK signaling drops, resulting in loss of BCL-2 expression, and rapid BIM-mediated apoptosis of myofibroblasts (36). Recently, it has been shown that pharmacological inhibition of BCL2-X_L_ can mimic this process and induce targeted BIM-mediated apoptosis in myofibroblasts and even revert established (murine) fibrosis (36).

In addition, in SSc skin, phosphatidylinositol 3-kinase (PI3K)/AKT serine/threonine kinase (AKT) signaling (37) is increased. This pathway facilitates myofibroblasts survival by inhibiting the activity of BAX. It does so by inactivating bcl2-associated agonist of cell death (BAD) via phosphorylation, after which this protein can no longer inhibit the function of anti-apoptotic proteins such as BCL2-X_L_. Many growth factors can induce PI3K/AKT signaling, including TGFβ. TGFβ signaling is increased in skin of SSc patients, and TGFβ has been demonstrated to induce AKT signaling in dermal fibroblasts to lower myofibroblasts' sensitivity for Fas-mediated apoptosis (34, 37, 38). Furthermore, TGFβ signaling also lowers expression of acid sphingomyelinase (SMPD1) (39). This enzyme induces the activation of protein phosphatase 2 (PP2A), i.e., an inhibitor of AKT signaling, and a reduction in SMPD1 thus enhances pro-survival AKT signaling. Additionaly, SMPD1 facilitates Fas-dependent apoptosis via its product; i.e., the lipid ceramide, which helps cluster Fas at the cell membrane, thus facilitating the formation of death inducing signaling complexes (40). In SSc fibroblasts, it has been shown that TGFβ lowers Fas-mediated apoptosis and that overexpression of SMPD1 prevented this effect, indicating its importance (39).

Finally, a role for micro RNAs (miRNA) in protecting myofibroblasts against apoptosis has been described in SSc. miRNAs are small non coding RNA molecules that can bind messenger RNAs and induce their degradation via an RNA-induced silencing complex (RISC). In SSc skin, expression of miRNA21 is increased, and this miRNA targets and degrades pro-apoptotic BAX mRNA (41). Additionally, miRNA21 targets phosphatase and tensin homolog (PTEN), which is an inhibitor of AKT signaling, as this phosphatase lowers intracellular PIP_3_ levels, the activator of AKT signaling (38). Via these mechanisms, presence of this miRNA lowers cellular sensitivity to apoptosis. Notably, TGFβ induces expression of miRNA21 in fibroblasts (38).

Together these mechanisms protect myofibroblasts from apoptosis in SSc which, in contrast to their final loss during wound healing, ensures their continued presence (long) after their formation.

## On the formation of myofibroblasts in SSc: pathways

**Figure 4:**
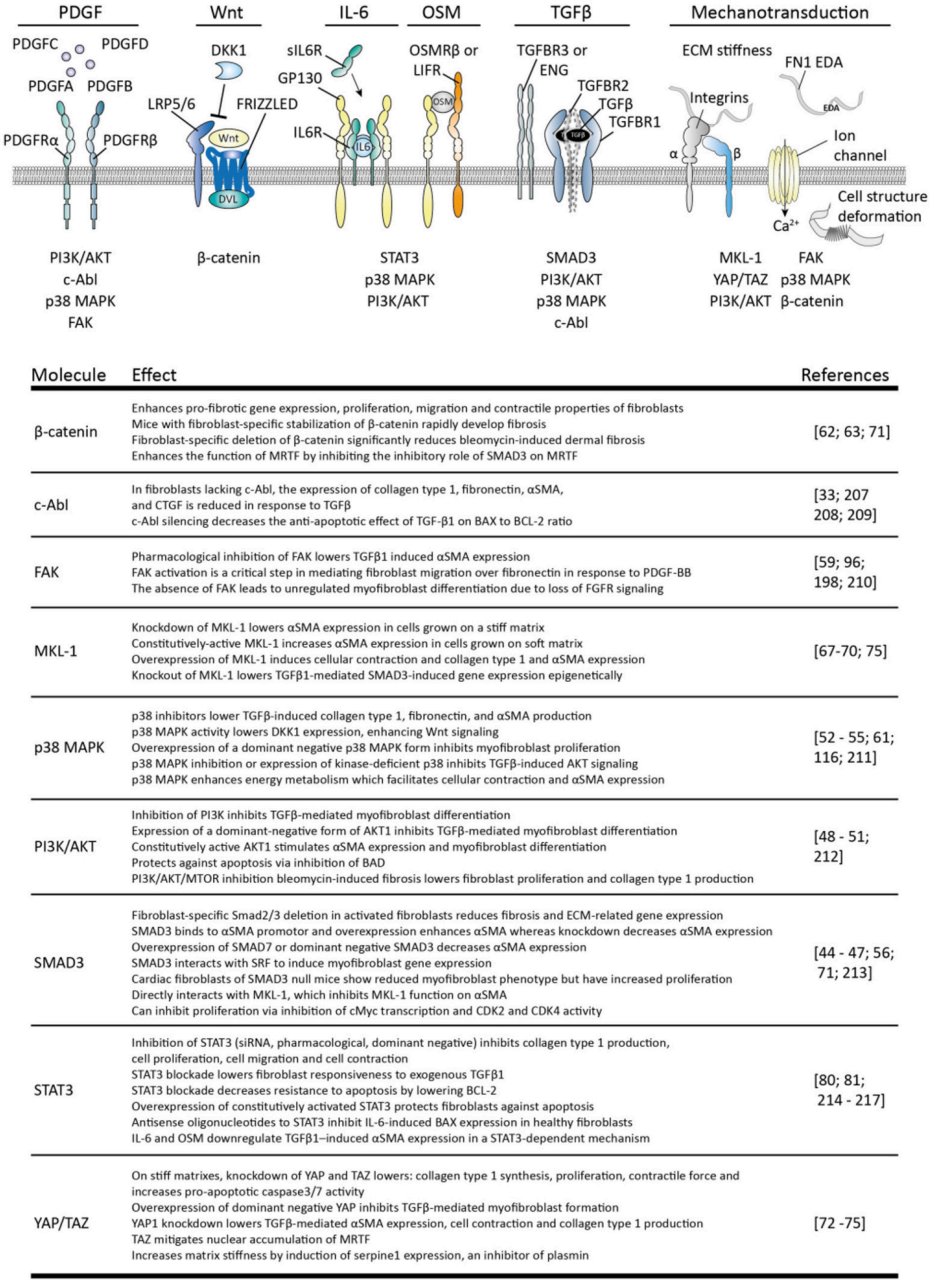
Stimuli for myofibroblast formation and their intracellular pathways. The four variants of platelet-derived growth factor (**PDGF**) can interact with homo- or heterodimers of PDGF receptor alpha (PDGFRα) and beta (PDGFRβ) to induce signaling by: phosphoinositide 3-kinase (PI3K)/AKT serine/threonine kinase (AKT), p38 mitogen-activated protein kinases (p38 MAPK), focal adhesion kinase (FAK) and tyrosine-protein kinase ABL1 (c-ABL). Canonical **Wnt** signaling is activated via formation of a wnt/frizzled/LRP5/6 complex which recruits disheveled (DVL) to the plasma membrane. This inhibits β-catenin degradation, leading to the accumulation of this protein and subsequent signaling. Interleukin 6 (**IL-6**) signaling uses a complex of membrane-bound or soluble IL-6 receptor (IL-6R) and glycoprotein 130 (gp130) to activate PI3K/AKT, p38MAPK and signal transducer and activator of transcription 3 (STAT3) signaling. Oncostatin M (**OSM**) also uses gp130, but together with oncostatin M receptor beta (OSMRβ) or leukemia inhibitory factor receptor (LIFR). Transforming growth factor beta (**TGF**β**)** induces heterotetramerization of TGFβ-receptor type I (TGFBR1) and II (TGBR2) and results in intracellular activation of SMAD3, p38 MAPK, PI3K/AKT c-ABL. TGFβ-receptor type III receptors such as betaglycan (TGFBR3), and endoglin (ENG) guide TGFβ availability and receptor complex formation. **Mechanotransduction** can occur via mechanosensitive ion channels, leading to e.g., calcium ion (Ca^2+^) influx, integrin complexes and deformation of cellular structures, leading to activation of myocardin-like protein 1 (MLK1), β-catenin, FAK, p38 MAPK, PI3K/AKT, and yes-associated protein 1 (YAP)/WW domain-containing transcription regulator protein 1 (TAZ). The effects of each of these pathways are listed in the table. Note that not all intracellular pathways are listed for each stimulus, only those connected to myofibroblast formation.

In SSc, not only the apoptosis of myofibroblasts is decreased but also their formation is increased. Myofibroblasts can originate in several ways, including the differentiation of fibroblasts toward myofibroblasts. This process is key in normal wound healing and facilitated by growth factors such as TGFβ, Wnts, damage associated molecular patterns such as fibronectin cloths, and tissue stiffness; the stiffer the matrix the more prone fibroblasts are to become myofibroblasts (42). In Figure 4 several intracellular pathways are listed that are involved in the transition of fibroblasts to myofibroblasts.

To begin, a key growth factor for myofibroblast formation is TGFβ; this growth factor directly induces extracellular matrix production and αSMA expression in fibroblasts. TGFβ activity is increased in skin of SSc patients, just as expression of its activating integrin αVβ5 (43, 44). This integrin can recognize latent TGFβ via its RGD domain and can mechanically separate the latency conferring peptides from the active peptide (42). The importance of integrin-mediated TGFβ activation is illustrated by the observation that inhibition of integrin αVβ5 by the use of antibodies or antisense RNA inhibits myofibroblasts formation (43, 44). Various intracellular pathways play a role in establishing the effects of TGFβ, in particular: SMAD3, PI3K/AKT, p38 MAPK, and c-ABL. Overexpression of SMAD3 enhances, whereas knockdown inhibits αSMA and extracellular matrix production in fibroblasts (45–48). Furthermore, fibroblast-specific deletion of SMAD3 reduces αSMA production and myofibroblast phenotype (49–52), for example, loss of SMAD3 lowers the number of activated myofibroblasts in cardiac fibrosis *in vivo* and reduces extracellular matrix production by myofibroblasts (47). Inhibition of PI3K/AKT signaling inhibits TGFβ-mediated myofibroblast formation, whereas overexpression of a constitutively active form of AKT1 enhances myofibroblasts development. The use of p38 MAPK inhibitors also lowers TGFβ-induced collagen type I and αSMA production and prevents TGFβ-induced AKT signaling (53–55). Additionally, this pathway alters cellular energy metabolism in such a way that is facilitates cellular contraction (56). Finally, in fibroblasts lacking c-ABL the expression of extracellular matrix molecules and αSMA is reduced in response to TGFβ. Of note, TGFβ can also negatively affect myofibroblasts. For example, SMAD3 can inhibit cellular proliferation via lowering the expression of c-myc and preventing the progression of cell division from G1 to S phase (57). Furthermore, pre-treatment of granulation tissue (myo) fibroblasts with TGFβ enhances their sensitivity to undergo bFGF-mediated apoptosis (58). This last observation illustrates that cellular context, e.g., the presence of bFGF, can greatly impact TGFβ signaling outcome.

Importantly, TGFβ facilitates the function of various other growth factors in fibroblasts. In SSc skin fibroblasts, TGFβ makes fibroblasts more sensitive to anabolic stimulation with platelet derived growth factor (PDGF), via induction of its receptor (PDGFR) (59). This growth factor induces extracellular matrix production and proliferation via the activation of PI3K/AKT, p38 MAPK, c-ABL, and focal adhesion kinase (FAK) pathways. In addition, this last pathway regulates PDGF-induced migration of myofibroblasts which recruits myofibroblasts to fibrotic areas (60). TGFβ and PDGF can work in concert, for example, in mouse corneal stromal fibroblasts co-stimulation of fibroblasts with TGFβ and PDGF greatly enhances myofibroblast formation compared to TGFβ alone (61).

Another pathway enhanced by TGFβ in SSc is canonical Wnt signaling. TGFβ signaling via p38 MAPK lowers dickkopf-1 (DKK1) expression (62), which is an inhibitor of canonical Wnt signaling via β-catenin. In skin and fibroblasts of SSc patients, decreased DKK1 expression is observed (62), together with increased β-catenin accumulation (63), and increased expression of Wnt signaling-related genes (62, 64). Stimulation of fibroblasts with canonical Wnts such as Wnt-1 or Wnt3a upregulates collagen type 1 and αSMA expression, and does so to a similar extent as TGFβ. Furthermore, mice with continuous fibroblast-specific Wnt signaling by artificial β-catenin stabilization rapidly develop skin fibrosis whereas fibroblast-specific deletion of β-catenin protects mice from bleomycin-induced skin fibrosis (63). Notably, Wnt signaling can induce autocrine TGFβ signaling (64) and overexpression of DKK1 protects mice even against TGFβ-receptor mediated skin fibrosis, indicating that both pathways are closely interwoven and interdependent.

Apart from the abovementioned effects, TGFβ can induce the expression of fibronectin 1 extra domain A (FN1 EDA) in (myo) fibroblasts. FN1 EDA is a splice variant of fibronectin which contains the so-called EDA domain. Normally, FN1 EDA is not expressed in healthy tissues but its expression is induced during wound healing (65) Fibroblasts can detect FN1 EDA via membrane bound receptors such as α4 containing integrins or toll like receptor 4 (TLR4), and its presence is a prerequisite for TGFβ-mediated myofibroblast formation; its expression precedes αSMA expression, and mice that lack their FN1 EDA domain are unable to produce myofibroblasts during injury (65–67). In turn, FN1 EDA facilitates the mechanical activation of TGFβ because it binds the latent form of TGFβ and presents this to integrins.

Next to these aforementioned stimuli, cellular mechanosensing is another crucial element in the transition of fibroblasts to myofibroblasts. Via for example intergrins, mechanosensitive ion channels, and cell structure deformation, fibroblasts can sense mechanical cues such as matrix stiffness. This mechanosensing results in activation of various intracellular pathways such as FAK, PI3K/AKT, p38 MAPK, and β-catenin, and activation of transcription activators such as myocardin-like protein 1 (MKL-1) and transcriptional coactivator YAP1 (YAP1) and WW domain-containing transcription regulator protein 1 (TAZ). Both MKL-1 and YAP/TAZ directly regulate myofibroblast phenotype. Knockdown of MKL-1 lowers αSMA expression in cells grown on a stiff matrix whereas overexpression of a constitutively active form of MKL-1 increases αSMA expression in cells grown on a soft matrix (68, 69). MKL-1 also activates collagen type 1 expression in lung fibroblasts (70). Furthermore, MKL-1 interacts with SMAD3 to bind the promoters of collagen type I and ASMA, and knockdown of MKL-1 lowers SMAD3-dependent gene expression (71). However, this interaction with SMAD3 can result in more rapid degradation of MKL-1, leading to repression of MKL-1-dependent genes (72). β-catenin has been shown to counteract this effect of SMAD3 (72), indicating that MKL-1 function depends on the integration of various pathways. Knockdown of YAP/TAZ in fibroblasts that are grown on stiff matrixes lowers proliferation, collagen type 1 synthesis, contractile force and increases pro-apoptotic caspase3 and caspase 7 activity. Furthermore, knockdown of YAP or overexpression of a dominant negative form lowers TGFβ-mediated myofibroblast formation (73–76). Notably, YAP/TAZ influence matrix stiffness by directly inducing serpine1 expression (73). Serpine1 inhibits the activation of plasmin, a protease which degrades extracellular matrix molecules such as fibrin and fibronection and can activate collagenases. Plasmin activity thus degrades and softens the extracellular matrix, but YAP/TAZ activity counteracts this (73) of note, serpine1 expression can also be rapidly and highly induced by TGFβ (77), and mechanical activation of TGFβ is enhanced in stiffer matrixes (42). Both YAP/TAZ and TGFβ activity can thus result in a feed forward loop in which tissue stiffness results in tissue stiffness-enhancing activity. Such a mechanism can explain continued fibrosis in absence of a exogenous stimulus.

Finally, the transition of fibroblasts to myofibroblasts is also facilitated by intracellular STAT3 signaling. STAT3 is induced by various cytokines such as interleukin 6 (IL-6) and oncostatin M (OSM). IL-6 expression is strongly expressed in SSc skin fibroblasts (78), and *in vitro*, stimulation of SSc skin fibroblasts with IL-6 results in collagen and αSMA expression (78–80). Furthermore, in the murine bleomycin model for skin fibrosis, knockout of IL-6 reduces skin pathology, as does administration of an anti-IL-6 receptor antibody (MR16-1) (79). In SSc skin, STAT3 signaling is activated (81) resulting in pro-fibrotic gene expression in fibroblasts; for example, STAT3 regulates collagen type I expression in SSc skin fibroblasts (82). However, of note, in lungs of SSc patients no enhanced STAT3 activation can be observed (82). Importantly, in both bleomycin induced skin and lung fibrosis in mice, knockout or pharmacological inhibition of STAT3 ameliorates fibrosis (83) (81). Furthermore, in both models, STAT3 was shown to be downstream of TGFβ signaling, as inhibition of STAT3 prevented TGFβ-induced myofibroblasts formation (81, 83).

Together these pathways can mediate the transition of fibroblasts to myofibroblasts and direct myofibroblasts activity after formation but cellular context plays an important role in guiding the outcome.

## On the formation of myofibroblasts in SSc: cells

**Figure 5:**
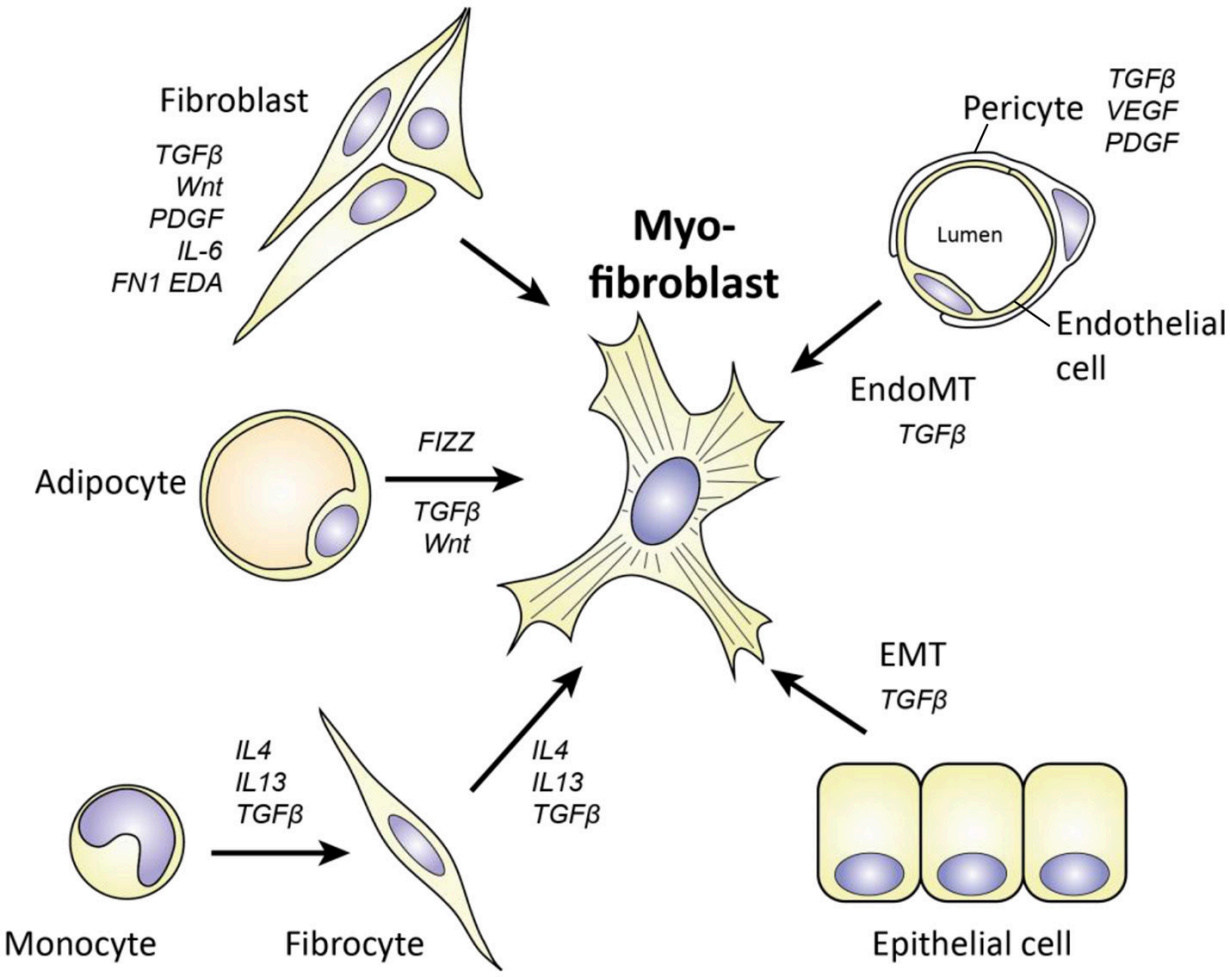
Cellular origins of myofibroblasts in SSc. Myofibroblasts can originate from various cell types, including fibroblasts, adipocytes, monocytes/fibrocytes, pericytes, endothelial cells, and epithelial cells. Key molecules for each transition are depicted. For epithelial cells to become myofibroblasts, they have to undergo epithelial to mesenchymal transition (EMT). For endothelial cells a similar process is needed, called endothelial to mesenchymal transition (EndoMT).

Apart from the transition of fibroblasts to myofibroblasts, an important source of myofibroblasts in SSc is the transdifferentiation of other cell types (Figure 5).

To begin, one cell type that can function as a source of myofibroblasts is the pericyte. These contractile cells surround endothelial cells in the microvasculature and regulate blood flow. Pericytes already express αSMA, and can become myofibroblasts if they leave their cellular niche and start to express proteins such as collagen type I and FN1-EDA. That this process occurs in SSc is suggested by a study that shows that pericytes in SSc skin, but not in healthy skin, express FN1-EDA and other myofibroblast markers (27). Furthermore, using lineage tracing it has elegantly been demonstrated that perivascular cells end up in skin scars as myofibroblasts (84). In addition, this transition is also observed in lung, liver, and kidney fibrosis (85), indicating that pericyte to myofibroblast transition is a common aspect of many fibrotic disorders. Putative drivers of this transition are VEGF, PDGF, and TGFβ.

Another cell type which can give rise to myofibroblasts is the fibrocyte. Fibrocytes are circulating cells of myeloid origin with stem cell like characteristics. These cells were first identified as the myeloid cells that rapidly invade wounds and, in contrast to other myeloid cells, produce ECM molecules. Their migration to wounds is guided by damage associated molecular patterns (DAMPs) and chemokines such as Chemokine (C-C motif) ligand 21 (CCL21) (86), and after arrival, these cells start differentiating into a myofibroblast-like phenotype under the influence of factors such as TGFβ (86). Of note, fibrocytes can originate from monocytes, and, importantly, SSc monocytes display increased maturation toward myofibroblasts as indicated by αSMA expression when compared to monocytes from healthy controls (87). Furthermore, fibrocyte presence and involvement in pulmonary fibrosis can readily be detected in SSc (87). Paradoxically, fibrocyte numbers in blood are lower in SSc patients than in healthy controls. Possibly, these cells are recruited out of the blood compartment into affected areas which would explain their lower numbers in blood.

In addition to the abovementioned cells, adipocytes, i.e., fat cells, are another source of myofibroblasts in SSc. Via the process of adipocyte to myofibroblast transition these cells can become myofibroblasts. In SSc skin, subcutaneous fat disappears over the course of the disease (88). With the use of adiponectin-lineage tracking, it has been demonstrated in the murine bleomycin model of skin fibrosis that adipocytes can lose their adipocyte-related gene expression and start expressing αSMA to become myofibroblasts (88). Importantly, in this model of skin fibrosis the loss of fat tissue precedes fibrosis (88) indicating that this process can underlie the fibrotic process. Adipocyte to myofibroblast transition is strongly driven by TGFβ (88), Found in inflammatory zone 1 (FIZZ1) and possibly Wnt signaling (89). *In vitro*, FIZZ1 suppresses adipogenesis and stimulates myofibroblast differentiation via Notch1 signaling. Furthermore, mice lacking FIZZ1 retain more fat and develop less fibrosis in response to bleomycin skin injury (90). Of note, FIZZ1 has also been attributed a role in lung fibrosis, by recruiting bone marrow derived stem like cells like to damaged lung tissue (91), and its levels are increased in serum of SSc patients (90).

Finally, two important sources of myofibroblasts in SSc are epithelial to mesenchymal transition (EMT) and endothelial to mesenchymal transition (EndoMT). In both processes, respectively epithelial and endothelial cells lose their phenotype and become myofibroblasts. Both processes can be observed in SSc. EndoMT can be identified using immunohistochemistry by observing endothelial cells with both endothelial (cluster of differentiation (CD31, and VE-cadherin) and myofibroblast markers (αSMA), and has been observed in skin and in lungs of SSc patients (92, 93). Furthermore, EndoMT has been linked to endothelial dysfunction as a cause for pulmonary arterial hypertension, a major complication in SSc (94). Notably, endothelial cells that undergo EndoMT produce more IL-6, IL-8 and TNFα compared to normal endothelial cells (94). EMT is an important driver of lung fibrosis, in which alveolar epithelial cells become myofibroblasts (95). This was demonstrated using alveolar specific lineage tracking, which visualized that alveolar cells started to express αSMA upon overexpression of TGFβ1 (95). The role of EMT in skin fibrosis is less clear. In SSc skin, expression of the key EMT inducing transcription factor SNAI1 can be observed in keratinocytes, but not loss of their epithelial E-Cadherin marker (96). Possibly, the EMT process is therefore only partially evoked here.

In conclusion, myofibroblasts can originate from many sources in SSc. Possibly, their origin has an effect on their phenotype and function, yet little is known if this is the case.

## On increased activity of myofibroblasts in SSc

Because of reduced apoptosis and increased formation, myofibroblasts numbers are increased in SSc. However, also their activity is markedly increased in SSc. For example, skin (myo) fibroblasts of SSc patients show more activation of focal adhesion kinase (FAK) *in vitro* than those of controls (97). This focal adhesion kinase is a key component of integrin signaling, and regulates fibroblast migration, survival and growth. Furthermore, *in vitro*, (myo)fibroblasts obtained from SSc patients produce more extracellular matrix molecules such as collagen type I than those of healthy controls, and their migratory and contractile properties are also increased (19, 98). Because the activated phenotype of SSc (myo) fibroblasts persists *ex vivo*, e.g., during cell culture, epigenetic changes most likely play an important role in this phenotype. For example, recent research has shown that in SSc skin fibroblasts, expression of the histone demethylase Jumonji domain-containing protein 3 (JMJD3) is increased (99). This histone demethylase removes the so-called H3K27me3 mark from histones, and this mark can repress expression of pro-fibrotic genes such as collagen type I in fibroblasts (100). Furthermore, pharmacological inhibition of H3K27 trimethylation induces skin fibrosis and aggravates pathology in bleomcyin induced skin fibrosis (100). A key target which is activated by JMJD3 is Fos-related antigen 2 (Fra-2) (99). This transcription factor has been identified as an important regulator of extracellular matrix production in skin fibroblasts; transgenic overexpression of Fra-2 results in increased dermal thickness and myofibroblast formation and is a mouse model for SSc (101), whereas knockdown of Fra-2 reduces both TGFβ- and PDGF-induced collagen production in primary skin fibroblasts of SSc patients (102).

Next to epigenetic changes, several cytokines can enhance the formation and function of myofibroblasts. In Table 1 an overview is given of how various cytokines affect myofibroblasts activity. As already mentioned TGFβ, PDGF, Wnts, IL-6, and OSM are key cytokines for myofibroblasts formation and activity. In addition to these factors, both IL-4 and IL-13 are pro-fibrotic (150). Both cytokines induce αSMA expression in primary lung fibroblasts in a dose- and time-dependent manner (105, 150), and enhance the production of collagen type I in normal fibroblasts (108). IL-22 has been described to have similar effect (118). Less clear is the role of IL-1 and Tumor necrosis factor α (TNFα). Of these factors both inhibitory and stimulatory effects on (myo) fibroblasts have been described. In atrial and intestinal myofibroblasts TNFα induces proliferation and collagen synthesis (119, 120). However, in dermal fibroblasts TNFα can inhibit αSMA expression by inhibiting TGFβ signaling (124). Interleukin 1 can not only induce, but also inhibit, collagen production, proliferation and myofibroblasts formation in dermal and lung fibroblasts by inhibition of TGFβ signaling (103, 104). Apart from these stimulatory cytokines, several signaling molecules inhibit myofibroblast formation and activity. For example, interferon γ (IFNγ) inhibits collagen synthesis, sensitizes dermal fibroblast to Fas-mediated apoptosis (125, 126) and inhibits IL-4 effects (125). Prostaglandin E2 has similar effects on formation and apoptosis in lung and keloid fibroblasts (145–147). The role of basic fibroblast growth factor (FGF2) is less clear, as it can inhibit TGFβ-mediated myofibroblast formation (140), but can also increase myofibroblast proliferation (151).

**Table 1:** Influence of various cytokines on myofibroblast biology.

**Signal molecule**	**Type of (myo)-fibroblasts**	**Observations**	**Effect**	**References**	**Remarks**
IL-1	Dermal, Lung	Stimulates collagen type 1 production Stimulates proliferation Inhibits collagen type 1 production Reduces formation and proliferation	+ + − −	(103) (103) (103, 104) (103, 104)	Can inhibit TGFβ effects
IL-4	Lung Lung Keloid, Dermal	Increases formation (αSMA expression) Increases proliferation Increases collagen type 1 production	+ + +	(105) (106, 107) (108)	Stimulates Th2 formation and alternative activation of macrophages
IL-6	Lung Lung Dermal	Inhibition of sIL6R signaling lowers myofibroblasts numbers Inhibition of sIL6R signaling lowers collagen and fibronectin deposition Increases collagen type I and αSMA expression	+ + +	(109) (109) (78, 110)	sIL6R signaling enhances TGFβ signaling (110)
IL-10	Dermal, cardiac Dermal Dermal	Reduces collagen type I production Reduces TGFβ and TNFα induced proliferation Lowers sensitivity to FAS-induced apoptosis	− − +	(111–113) (114)	Induces TGFβ production by macrophages
IL-13	Lung Lung Keloid & Dermal	Increases αSMA expression Increases proliferation Increases collagen type 1 production	+ + +	(105) (105, 107) (108)	
IL-17	Dermal Lung	Inhibits collagen type 1 production Stimulates collagen, TGFβ and IL-6 production	− +	(115, 116) (117)	Induces IL-6 production and immune cell attraction in fibroblasts
IL-22		Induces differentiation Induces collagen type 1 production No effect on collagen production	+ + =	(118)	Enhances fibroblast response to TNFα
TNFα	Intestinal Intestinal, Dermal, cardiac Palmar dermal Dermal Lung, Dermal Dermal	Induces collagen accumulation via TNFR2 Induces proliferation via TNFR2 Induces myofibroblasts formation Induces apoptosis via TNFR1 Sensitizes fibroblasts to FAS-induced apoptosis Suppresses αSMA expression and TGFβ effects	+ + + − − −	(119) (119–121) (119) (122) (114, 123) (124)	Alters PDGF signaling (121)
IFNγ	Dermal Dermal Dermal	Inhibits collagen synthesis Sensitizes to FAS-induced apoptosis Inhibits proliferation in fast dividing cells, stimulates proliferation in slowly dividing cells	− − ?	(125, 126) (114)	Antagonizes IL-4 (125) and TGFβ (127)
OSM	Lung Lung, Dermal Lung, Dermal Lung	Increases αSMA expression and contraction Increases proliferation Increases collagen production Increases cell survival	+ + + +	(128) (129, 130) (117)(131) (117)	OSM signaling is augmented by IL4 or IL13 (132)
CCL2	Lung	Inhibits apoptosis via production of IL-6	+	(133)	Chemoattractant of monocytes Stimulates IL-4 production in Th2 cells
TGFβ	Lung, Dermal, cardiac, keloid	Increases αSMA expression Stimulates collagen type 1 production Stimulates proliferation Increases contraction Inhibits apoptosis Stimulates apoptosis Inhibits proliferation	+ + + + + − −	(38, 45–48, 134, 135) (136) (58) (57)	Works in concert with Integrin-FAK Enhances Wnt signaling in SSc by downregulating DKK1 via p38 MAPK (62) Causes hyperactivation of STAT3 in SSc (81) Counteracted by bFGF signaling
CTGF	Corneal	Facilitates TGFβ effects	+	(137)	
PDGF	Corneal, Dermal, Lung	Increases αSMA expression Stimulates collagen type 1 production Stimulates proliferation	+ + +	(61, 138, 139)	TGFβ stimulates PDGFR expression (59)
FGF2 (bFGF)	Dermal Dermal	Inhibits TGFβ-induced myofibroblasts formation Increases fibroblast proliferation Stimulates apoptosis	−	(140) (140) (58)	
Wnt		Canonical Wnt signaling induces fibroblast proliferation and migration, collagen gel contraction, and myofibroblast differentiation	+	(62–64, 89)	Induces TGFβ production (64)
Histamine	Lung Dermal Dermal	Enhances proliferation via a H2R Increases αSMA expression Inhibits TGFβ-induced αSMA expression via H1R	+ + −	(141) (142) (143)	
Leukotriene D4	Lung	Enhances TGFβ-induced collagen synthesis	+	(144)	
PGE2	Lung Keloid Lung	Induces apoptosis Inhibits migration, contraction and TGFβ-induced collagen synthesis Inhibits myofibroblasts formation	− − −	(145) (146) (147)	
Serotonin	Lung Lung	5-HT_2B_ receptor antagonists reduce myofibroblast differentiation Induces extracellular matrix synthesis	+ +	(148) (149)	Effects depend on TGFβ signaling (149)

The increased presence and activity of myofibroblasts in SSc results in various deleterious effects. First of all, their excessive matrix production and remodeling capabilities can destruct organ architecture leading to loss of function like in lung fibrosis. Furthermore, deposition of extracellular matrix molecules such as collagens in the interstitial space of lung tissue inhibits gas exchange, greatly lowering lung function and resulting in interstitial lung disease. In skin excessive matrix deposition increases stiffness, increases hardness, and leads to loss of cutaneous tissues like, fat tissue, sweat glands, hair follicles, and sebaceous glands (152). In the gastro-intestinal tract, myofibroblast-induced fibrosis negatively affect motility, digestion, absorption, and excretion (153).

Blood vessel function is also impacted by myofibroblasts. To begin, myofibroblasts produce endothelin-1 (15). Endothelin 1 is a potent vasoconstrictor, leading to increased blood pressure. Notably, endothelin 1 also stimulates the formation of new myofibroblasts. Furthermore, myofibroblasts also produce VEGF (154), e.g., during wound healing, and can also express angiopoietin 1 and 2, both of which stimulate the formation of new blood vessels (155). As mentioned, myofibroblasts also produce and activate TGFβ. VEGF, angiopoietins, and TGFβ are all key regulators of endothelial homeostasis, and normally these factors are well balanced to maintain this homeostasis. However, this balance can be disturbed by the myofibroblast's production of these factors, leading to aberrant vascular remodeling. For example, uncontrolled VEGF signaling has been suggested to be a cause for capillary malformations in SSc (154).

Myofibroblast also have an immunomodulatory role. As mentioned, they express for example interleukin 1 (IL-1), interleukin 6 (IL-6), interleukin 8 (IL-8), monocyte chemoattractive protein 1 (MCP-1) (13). Both IL-8 and MCP-1, also known as CCL2, are chemokines, attracting neutrophils, monocytes and T cells and in this way facilitate inflammation. Both IL-1 and IL-6 can enhances pro-inflammatory gene expression in immune cells. Furthermore, both factors can participate in the differentiation of monocytes toward macrophages and play a role in the differentiation of naive T-cells toward an effector subtype (156).

## On the role of the (innate) immune system in myofibroblast formation and function

**Figure 6:**
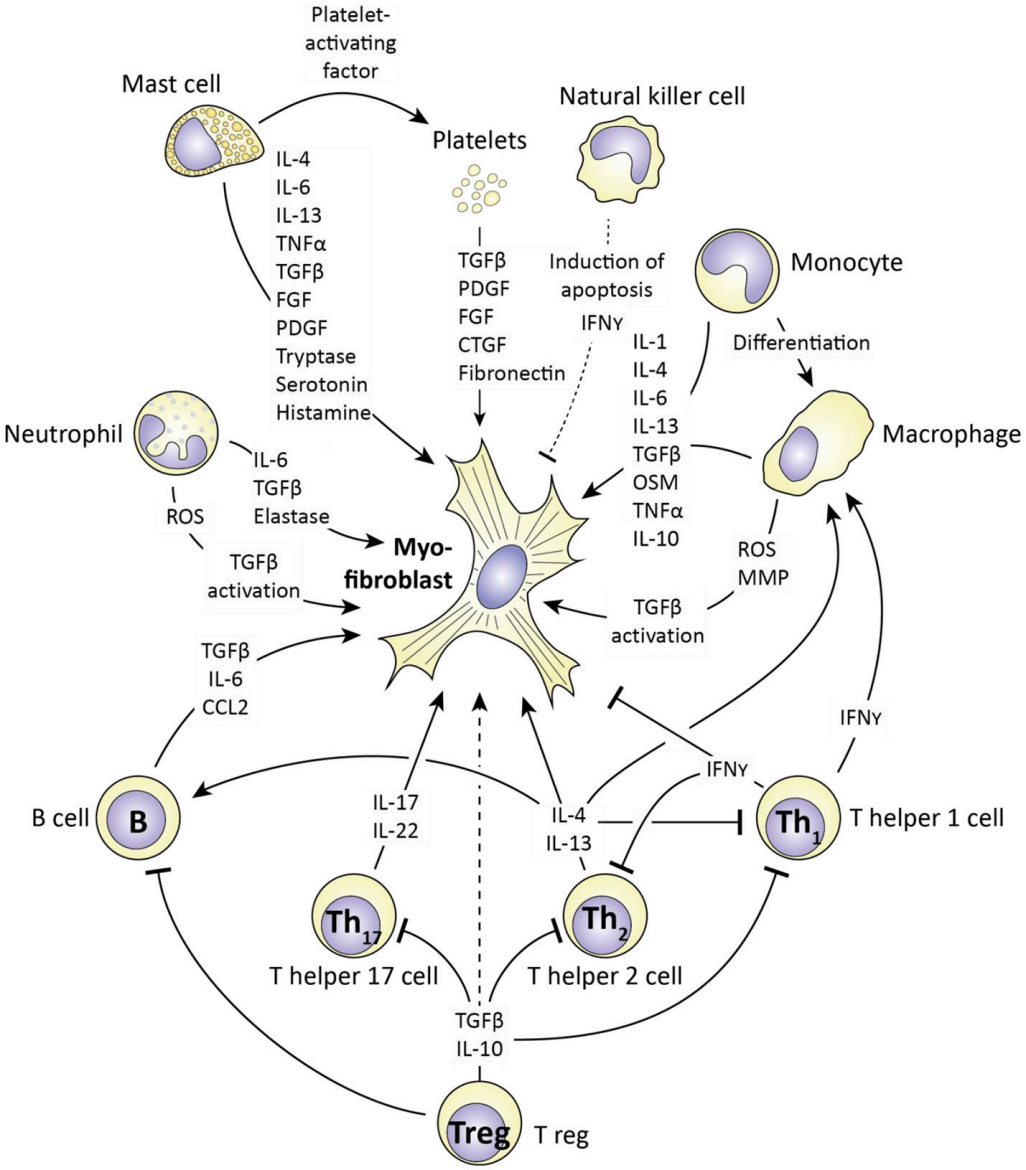
The influence of immune cells on myofibroblast formation and function. Immune cells produce various mediators (also see Table 1) that influence myofibroblast formation and function. For each cell type (and platelets) the corresponding mediators are depicted. Cells which stimulate myofibroblast function include mast cells, monocytes/macrophages and T helper 2 lymphocytes via e.g. production of IL-4, IL-13, and TGFβ. In contrast, T helper 1 cells can negatively affect myofibroblast function via production of interferon gamma (IFNγ). Importantly, the ultimate outcome of an immune response on myofibroblast function depends on the interplay between immune cells, as this interplay regulates the production of the mediators the affect myofibroblast function.

Myofibroblast survival, formation, and function are all increased in SSc. The (innate) immune system plays an important role in this. In Figure 6 an overview is given of how.

One immune cell which can induce myofibroblasts formation and activity is the mast cell. Mast cells are part of the innate immune system and well known for their role in allergy. However, they have already been implicated in SSc pathophysiology for a long time (157), because they can produce several mediators which stimulate fibrosis (158). One such factor is Platelet-activating factor, which stimulates platelet aggregation and degranulation. Platelet degranulation releases many (growth) factors, including TGFβ, PDGF, and fibronectin, all of which are factors which stimulate myofibroblasts formation and function. Another product of mast cells and platelets is serotonin. Serotonin has long been implicated in fibrotic disorders; already in 1958 it was demonstrated that subcutaneous injections of serotonin induce skin fibrosis (159). More recently, it was demonstrated that serotonin directly increases extracellular matrix production in primary skin fibroblasts (149). This effect runs via the 5H-T_2b_ receptor; inhibition of this receptor with terguride decreases collagen and fibronectin production by fibroblasts. Importantly, mice that lack this receptor (${5\text{H}-\text{T}}_{2\text{b}}^{-/-}$) are protected against bleomycin-induced skin fibrosis, just as mice in which the 5H-T_2b_, receptor is pharmacologically inhibited (149). Mast cells also produce tryptase, a serine proteinase, which, remarkably, stimulates fibroblast proliferation and collagen production (142, 160, 161), and histamine, which also induces (lung) fibroblast proliferation (141). Next to these factors, mast cells also produce a large array of pro-fibrotic cytokines; IL-4, IL-6, IL-13 TNF-α, TGFβ, and PDGF (158) which directly stimulate the formation and activity of myofibroblasts. Interestingly, mast cells can directly interact with skin (myo) fibroblasts, and this facilitates their role in fibrosis. This interaction was shown to be serpine1 dependent. Apart from the aforementioned role as inhibitor of plasmin activation, this protein is a chemotactic for mast cells and induces the expression of intercellular adhesion molecule 1 (ICAM1) in fibroblasts, which is needed for mast cells to adhere to fibroblasts (162). Of note, serpine1 is a downstream target of TGFβ signaling in many cell types, including fibroblasts.

Another innate immune cell which can have a pro-fibrotic role is the neutrophil. Like mast cells, neutrophils produce various pro-fibrotic cytokines including: TGFβ, IL-6, and VEGF (163). Furthermore, activated neutrophils release reactive oxygen species (ROS) (164). Reactive oxygen species activate fibroblasts and stimulate fibrosis (165). In part, this effect is due to the activation of TGFβ. Chemical reaction of reactive oxygen species with latent TGFβ disrupts the quaternary protein structure of latent TGFβ, and results in release of active TGFβ (165). Of note, neutrophils of SSc patients release more ROS than neutrophils of healthy controls when challenged with TNFα (164). Recently, it was also demonstrated that neutrophil elastase, a serine proteinase, can induce myofibroblasts formation (166). Mice lacking this enzyme are protected against asbestos-induced lung fibrosis, and *in vitro* neutrophil elastase directly stimulates myofibroblasts formation, proliferation, and contractility (166). Furthermore, pharmacological inhibition of neutrophil elastase by sivelestat protects mice from bleomycin induced lung fibrosis (167), demonstrating that at least in lungs, neutrophil elastase is pro-fibrotic.

Next to mast cells and neutrophils, also macrophages can stimulate the formation and activity of myofibroblasts. To begin, macrophages, and their precursor the monocyte, can produce large amounts of TGFβ, for example during bleomycin induced lung fibrosis in rats (168). Apart from TGFβ, macrophages produce many cytokines with pro-fibrotic effects, including IL-4, IL-6, and IL-13 (156). Especially alternatively activated macrophages, also known as M2 macrophages, are associated with production of pro-fibrotic cytokines. These cells have a less pro-inflammatory and more repair oriented phenotype than classically activated macrophages, i.e., M1 macrophages (156). Macrophages, like neutrophils, also produce reactive oxygen species which enhance fibrosis. The importance of macrophages in regulating fibrosis is demonstrated by the observation that in mice, deletion of lung macrophages using liposomal chlodronate reduces bleomycin induced lung fibrosis, and a similar effect is obtained if circulating monocytes are depleted using liposomal chlodronate (169).

A cell of the innate immune system with a possible anti-fibrotic role is the natural killer (NK) cell. In liver fibrosis, this cell type can recognize myofibroblasts and stimulate them to undergo apoptosis (170). Furthermore, NK cells produce IFNγ a strong inhibitor of myofibroblasts formation and function (171). However, in SSc, both the killing ability and stimulation-dependent IFNγ production of NK cells has been reported to be reduced (171).

In addition to the cells of the innate immune system, cells of the acquired immune system also play a role in fibrosis. A cell type particularly associated with fibrosis in SSc is the T helper 2 cell (Th_2_). These cells produce the pro-fibrotic cytokines IL-4, IL-5, and IL-13, which directly stimulate fibroblasts but also induce the formation of alternatively activated macrophages (172, 173). SSc is characterized by Th_2_ polarization, i.e., a Th_2_ cytokine profile in blood, and importantly, in SSc, the extent of Th_2_ polarization directly positively correlates with active interstitial lung disease (i.e., lung fibrosis), supporting for a role of Th_2_ cells in this process (132). Also T helper 17 cells (Th_17_) can play a role in fibrosis, in part via their production of IL-17 and IL-22, which can stimulate collagen, TGFβ and IL-6 production in pulmonary fibroblasts (117, 118). In contrast to these two T helper subtypes, T helper 1 (Th_1_) cells are more associated with inhibition of myofibroblast function, for example in pulmonary fibrosis (174). This effect of Th_1_ cells is attributed to their production of IFNγ, which directly inhibits myofibroblast formation and function, but also directs macrophage polarization away from the pro-fibrotic, alternatively activated (M2) phenotype. The role of regulatory T cells (T_reg_) in fibrosis and myofibroblast activity is less clear. These cells produce TGFβ and IL-10 which can directly regulate myofibroblast function, but also affect the activity of Th_1_, Th_2_, and Th_17_ cells. Because these effector T cells have different functions on myofibroblasts, the end effect of T_reg_ activity is difficult to predict. Finally, B lymphocytes have also been demonstrated to be able to promote fibrosis; co-culture of B cells with skin fibroblasts induced the expression of collagen and αSMA by the latter (175). For this effect cell-cell contact was required, as the use of a transwell system negated the stimulatory effect of B cells on fibroblast activity (175). However, B lymphocytes are able to produce TGFβ, CCL2, and IL-6, which enhance myofibroblast activity without the need for cell contact.

## On therapeutic targeting of myofibroblasts in SSc

In view of the role of myofibroblasts in SSc, inhibiting their formation or function makes an excellent option for targeted therapy. Several compounds that have been investigated in, or are currently under investigation in clinical trials are listed in Tables 2, 3, respectively. Whether these compounds truly target myofibroblasts is up for debate, yet they do target cellular processes important for myofibroblast formation and function.

**Table 2:** Clinical trials conducted with putative anti-fibrotic agents in SSc.

	**Target**	**Type of trial**	**Phase**	**Duration (months)**	**Number of patients**	**Type of patients**	**Result**	**References**
Abatacept	CD80/ CD86	Randomized, double-blind, placebo-controlled	I/II	6	10	dcSSc	Five out of seven patients (71%) randomized to abatacept and one out of three patients (33%) randomized to placebo experienced ≥30% improvement in skin score	(176)
Bovine Collagen type I		Randomized, double-blind, placebo-controlled	II	12–15	168	dcSSc 6 months stable mRSS of ≥16	No significant differences in the mean change in MRSS or other key clinical parameters between the CI and placebo treatment groups at 12 or at 15 months	(177)
C-82 topical gel	CBP β catenin	Randomized, double-blind, placebo-controlled	I/Ii	1	17	dcSSc ≤3 years, increase in mRSS ≥ 5 in 6 months	No detected result in clinically efficacy on mRSS	(178)
Dasatinib	PDGFR c-ABL	Single-arm, open label	I/II	9	31	dcSSc ≤3 years, mRSS ≥15	No significant clinical efficacy on mRSS or pulmonary function test	(179)
Fresolimumab	TGFβ	Single-arm Open label	I	6	15	dcSSc ≤2 years mRSS ≥15	Improved mRSS Reduced TGFβ dependent gene expression in skin biopsies	(180)
Imatinib	PDGFR c-ABL	Single-arm Open label	IIa	6	24	dcSSc	Improved skin morphology and mRSS compared to baseline	(181)
		Randomized Double-blind Placebo-controlled	II	6	28	mophea >20% or SSc with mRSS >20/51	This study failed to demonstrate the efficacy of imatinib on mRSS	(182)
		Randomized Double-blind Placebo-controlled	II	6	10	active dcSSc	Imatinib was poorly tolerated; only 10 of 20 patients included	(183)
		Single-arm Open-label	II	6	26	SSc patients with active pulmonary involvement and unresponsive to cyclophosphamide	Stabilized lung function, no effect on skin	(184)
Metelimumab (CAT 192)	TGFβ	Randomized Double-blind Placebo-controlled	I/II	6	45	SSc duration of <18 months	No evidence of a treatment effect	(185)
Nilotinib	PDGFR c-ABL	Single-arm Open label	IIa	6 and 12	10	dcSSc ≤3 yr mRSS ≥16	Significant MRSS improvement	(186)
Pirfenidone	?	Randomized Open-label	II	4	63	SSc <7 years	No clinically relevant differences on skin on FVD	(187)
Relaxin	Relaxin receptor	Randomized Double-Blind Placebo-controlled	II	6	231	dcSSc ≤5 years mRSS ≥16	Recombinant relaxin was not significantly better than placebo in improving total skin score, pulmonary function, or functional disability in	(188)
Tocilizumab	IL6 receptor	Double-blind, placebo-controlled	II	12	87	progressive SSc ≤5 yr 15 ≥ mRSS ≤40	Not associated with a significant reduction in skin thickening	(189)
		Open label extension phase		24	51		Skin score improvement and FVC stabilization	(190)

**Table 3:** Clinical trials currently underway with putative anti-fibrotic agents in SSc.

**Compound**	**Target**	**Type of trial**	**Phase**	**Duration (months)**	**Number of patients**	**Type of patients**	**Identifier**
Dabigatran	Thrombin	Single-arm open label	I	6	15	SSc <7 yr with ILD	NCT02426229
GSK2330811	OSM	Randomized Double-blind Placebo-controlled	II	3	40	active dcSSc <5 years 10 ≥ mRSS ≤35	NCT02453256
Lenabasum (CT-101)	CBr2	Randomized double-blind Placebo-controlled	III	12	354	dcSSc <6 yr	NCT03398837
Nintedanib	PDGFR/VEGFR/FGFR	Randomized double-blind Placebo-controlled	III	12 up to 24	580	SSc <7 yr with ILD	NCT02597933
SAR156597	IL4-13	Randomized double-blind Placebo-controlled	II	6	94	dcSSc	NCT02921971
Tofacitinib	JAK1/JAK3 kinase	Randomized double-blind Placebo-controlled	III	12 (+ 12 open label)	212	dcSSc <5 years 10 ≥ mRSS ≤35	NCT02453256

To begin, one compound that is currently under investigation is tocilizumab. Tocilizumab is a humanized antibody directed against the IL-6 receptor and currently enrolled in a phase III trial for SSc therapy (98). In an initial phase 2 double-blind, placebo controlled study tocilizumab did not significant reduce skin thickening (189), but the open label extension phase of this study did show encouraging protective effects on skin thickening and loss of forced vital capacity in SSc patients (190). Interestingly, skin biopsies were collected from enrolled patients before and after treatment and analyzed for fibroblast phenotype. Treatment with tocilizumab for 24 weeks decreased fibroblast protein production, migration and contractility compared to baseline (98). Furthermore, a large effect between the tocilizumab and placebo treated groups was observed on gene expression profile; in the placebo group, gene expression was not significantly altered over 24 weeks, whereas in the tocilizumab treated group 2,136 genes were significantly differentially expressed. Strikingly, many TGFβ signaling related genes, together with cell contractility pathways, were downregulated to a level similar to normal expression levels (98). This study thus demonstrates that tocilizumab is a serious candidate for targeting (myo-) fibroblasts in SSc.

In view of these results with tocilizumab, the results of tofacitinib in SSc treatment will be of interest. Tofacitinib is a small molecule JAK1 and JAK3 kinase inhibitor downstream of IL-6 signaling which can be used for the treatment of rheumatoid arthritis. Because JAK1 and JAK3 both activate STAT3 this compound can be expected to inhibit myofibroblast function. Currently, tofacitinib is under investigation in a small double-blinded phase I/II trial for safety and efficacy in SSc.

Another compound of interest for treatment of fibrosis in SSc is pirfenidone. Pirfenidone is used for the treatment of idiopathic pulmonary fibrosis and is a pyridone derivative. Dietary intake of this compound was shown to inhibit bleomycin-induced lung fibrosis in hamsters (191). Furthermore, this compound reduces fibroblast proliferation and attenuates TGFβ-induced αSMA and collagen production in primary skin fibroblast (192, 193). In lung fibroblast of SSc patients with interstitial lung disease (ILD), treatment with pirfenidone lowered αSMA and fibronectin expression (194). However, in an open label phase 2 study with 63 SSc patients with ILD, no beneficial effects of pirfenidone were observed on disease outcomes (187).

Nintedanib is a small molecule kinase inhibitor of platelet derived growth factor receptor (PDGFR), vascular endothelial growth factor receptor (VEGFR), and fibroblast growth factor receptor (FGFR), which has been approved for the treatment of interstitial lung disease, and which can possibly be used for the treatment of (ILD in) SSc. For this latter application, it was recently granted a fast track designation by the U.S. Food and Drug Administration (FDA). In lung fibroblasts *in vitro*, nintedanib inhibits proliferation and motility as induced by FGF and PDGF, but also inhibits TGFβ-induced collagen deposition (195). *In vivo*, nintedanib protects mice and rats against bleomycin-induced lung fibrosis (195, 196), and lowers the amount of lymphocytes and neutrophils but not macrophages in bronchoalveolar lavage liquid in this model. In SSc skin fibroblasts, nintedanib also prevents proliferation and motility and lowers the expression of αSMA (197). Furthermore, it lowers the myofibroblasts count and skin fibrosis in bleomycin induced skin fibrosis in mice (197). In two phase 3 trials with patients suffering from interstitial lung disease, nintedanib profoundly lowered the decline in forced vital capacity (195, 198). Currently a phase 3 trial is underway to test its safety and efficacy in SSc.

PDGFR signaling is also targeted by nilotinib. This small molecule kinase inhibitor inhibits both PDGFR signaling and c-ABL signaling. In dermal fibroblasts, nilotinib inhibits TGFβ- or PDGF-induced collagen production in a dose dependent manner (199). Furthemore, this compound strongly lowered myofibroblasts formation and dermal thickness in bleomycin induced skin fibrosis in mice (199). Nilotinib has been tested in a small open label trial with 10 SSc patients (186), and has shown promising results on the modified Rodnan skin score after 6 and 12 months of treatment. A compound similar to nilotinib is imatinib, which also targets PDGFR and c-ABL signaling (200) This compound also reduces collagen production in skin fibroblasts, and also protects mice against bleomycin induced fibrosis via reduction of myofibroblasts formation and matrix deposition (200). Several clinical phase 2 trials have been conducted with imatinib in SSc. In an open label, single-arm clinical trial a positive effect of imatinib on skin thickening was reported after 6 months of treatment (181). However, in another placebo controlled, double blinded phase 2 study no efficacy on modified Rodnan skin score was reported after 6 months of treatment (182). Furthermore, a single center randomized, double-blind, placebo-controlled phase 2 trial warned for poor drug tolerance in SSc patients (183). A low dose of imatinib has also been tested in an open label study for treatment of intestitial lung disease in SSc patients unresponsive to cyclophosphamide (184), and was reported to stabilize lung function but again had no effect on skin.

In view of its pro-fibrotic effects, TGFβ has also been targeted in SSc. Currently, several TGFβ signaling targeting drugs are under clinical development for e.g., cancer treatment (201), but no trials for SSc are currently reported. In a small open label single center study, Fresolimumab (180), a high affinity TGFβ inactivating monoclonal antibody was recently tested in SSc patients, and reduced TGFβ dependent gene expression in skin biopsies and improved modified Rodnan skin score (180). In the past, a low affinity antibody had no such effect (185). Interestingly, several compounds that inhibit TGFβ activation by integrins are under development for various diseases. In a genetic mouse model for SSc (characterized by a mutation in fibrilin) antibodies against integrin α5β1 and integrin α5β3 inhibit skin fibrosis (202). The effects of these antibodies are mimicked by a TGFβ inhibiting antibody, illustrating that these effects possibly run via inhibition of TGFβ activation. In addition, a monoclonal antibody targeting integrin αVβ6 has been shown to protect mice from radiation induced fibrosis (203).

Currently also under development for treatment of SSc is lenabasum (CT-101). Lenabasum is a canabinoid type 2 receptor (CBr2) agonist and is currently being tested in a phase 3 trial for its efficacy and safety in treatment of SSc. Skin fibroblasts express CBr2, and this expression is increased in SSc (204). Stimulation of SSc skin fibroblasts with the synthetic cannabinoid WIN55,212-2 lowers matrix production, myofibroblast formation, and production of TGFβ, CTGF, and IL-6 (204). Furthermore, addition of this compound to mice inhibits bleomycin induced skin fibrosis by lowering fibroblast to myofibroblast transition and TGFβ, CTGF, and PDGF production (205). In addition, in bleomycin lung fibrosis activation of canabinoid receptor type 2 signaling by JWH133 lowered both the inflammatory response and extracellular collagen deposition, which was accompanied by reduced levels of TGFβ in blood (206). These observations make lenabasum a promising compound.

Finally, a therapy currently under investigation in a phase 2 trial as targeted therapy for SSc is the use of abatacept. Abatacept is a fusion protein consisting out of an IgG1 Fc tail fused with the extracellular part of CTL4 and is currently in use for the treatment of rheumatoid arthritis. Abatacept targets and prevents the function of CD80/CD86 molecules of professional antigen producing cells. This prevents these antigen presenting cells from activating T cells, as CD80/CD86 provide the co-stimulatory signal required in addition to MHCII binding to initiate T (helper) cell differentiation. Early SSc skin is characterized by perivascular T cell infiltrates (172), and (late stage) SSc patients have increased T_h2_ cell activation (T_h2_ polarization) and these cells express the pro-fibrotic cytokines IL-4, IL-5, IL-6, and IL-13 (172). In bleomycin induced skin fibrosis in mice, abatacept lowers the influx of monocytes, T cells and B cells into lesional areas, lowers IL-6 and IL-10 levels and lowers skin fibrosis (207). Importantly, abatacept does not affect skin fibrosis in the murine Tsk1 model of SSc which is less dependent on inflammation nor in bleomycin induced skin fibrosis in SCID mice which lack T cells. In a very small double-blind placebo controlled trial of 10 patients, abatacept improved the mRSS of patients. Especially patients with an inflammatory gene expression profile in their blood responded well to abatacept (176). These results indicate that abatacept can possibly eleviate inflammation driven fibrosis, but not by directly targeting myofibroblasts.

## Conclusion and future perspectives

In this review, we have addressed the role of myofibroblasts in SSc pathophysiology. The presence and formation of these cells are increased in SSc, giving rise to pathology due to their ability to produce excessive amounts of extracellular matrix molecules like collagen type I, their ability to affect vascular biology by production of e.g., VEGF and ET1, but also due to their immuno-modulatory effects via production of IL-6 and TGFβ. Targeting these cells is therefore a feasible strategy to get to a targeted therapy for SSc. Currently multiple drugs doing just that are in phase 3 trials, giving hope for the future of SSc treatment.

## Author contributions

All authors listed have made a substantial, direct and intellectual contribution to the work, and approved it for publication.

### Conflict of interest statement

The authors declare that the research was conducted in the absence of any commercial or financial relationships that could be construed as a potential conflict of interest.
